# Impact of physical factors on bio-control potential of *Lawsonia inermis* leaf extract and bio-formulations as fungicides

**DOI:** 10.1016/j.bbrep.2022.101361

**Published:** 2022-10-09

**Authors:** Sanjeev Meena, Pushpa Gehlot, Bhanu Raj Meena, Tripta Jain, Kanika Sharma

**Affiliations:** Microbial Research Laboratory, Department of Botany, University College of Science, Mohanlal Sukhadia University, Udaipur, India

**Keywords:** *Lawsonia inermis*, *Alternaria alternata*, Bio-formulation, Antifungal activity, Crop protection, Thermal stability

## Abstract

The present study is carried out to ascertain the effect of different physical factors (sunlight, temperature, pH) and storage conditions on the antimicrobial efficacy of *Lawsonia inermis* leaf extracts and bio-formulation against the *Alternaria alternata*. In addition, the phytotoxic potential of 100% alcoholic crude extract as well as the acetone fraction of young leaves of *Lawsonia inermis* was also checked on seed germination of chilli (*Capsicum annuum*). Results showed that there was no adverse effect of wet heat (50–100 °C) and dry heat (40–90 °C) on extract and bio-formulation efficacy. Storage for 6 and 12 months had no adverse effect on extract and bio-formulation efficacy and the antifungal activity was observed similar to freshly prepared extract. We have used concentrations of 5,10, 15, 20 and 25 mg/ml to perform a phytotoxicity assay. The measurement of phytotoxicity was done by using the Standard blotter method and the result revealed that 5, 10 and 15 mg/ml concentration of the extract was non phytotoxic and were further used for *in vivo* experiments. These plant extracts and bio-formulations have extensive antimicrobial potential to be explored for application in sustainable agriculture.

## 1 Introduction

Secondary metabolites present in the plants are directly related to their antimicrobial potential and can be affected by various physical factors [[1], [2], [3]]. These secondary metabolites are responsible for changes in microbial biochemistry and cytology, and saponins have been shown to limit fungal growth [4]. Excessive heating during the extraction process might disrupt physiologically active secondary metabolites in the plant extract, affecting their activity [5,6]. *Lawsonia inermis* (Henna) is a shrub or small tree that is grown as an ornamental and commercial dye crop in many parts of the world [7]. It is mostly found in Africa's tropics, subtropics, and semi-arid zones (tropical Savannah and tropical dry zones), as well as in South Asia and North Australia [8]. *Lawsonia inermis* belongs to the family Lythraceae. Its leaves were traditionally used to create red or black colouring on hands, feet, and hair for a variety of occasions, including weddings and religious festivals [9,10].

Physicochemical in plants are affected by a variety of physical conditions that alter their chemical composition and activity, as well as their antifungal potential [11]. The bioactive components of plant-based pesticides alter the biochemistry and cytology of microbes, thus preventing their propagation and disease spread. Alterations in temperature, pH, exposure to sunlight, and storage duration can affect the heterogeneous nature of biologically active plant metabolites which might change their respective biological activity [12].

The sustainability of their antifungal property in the presence of different physical factors is important to establish their commercial utility. Resistance of plant-based extracts and their bio-formulations against different physical factors has been evaluated by various investigators [13]. However, the extent of alteration in biological activities depends upon the type of biomaterial selected for the study, its phytochemical composition, the intensity of exposure and its source plant.

*Alternaria alternata* the causative agent of leaf spot disease in chilli is a wide spectrum ubiquitous necrotrophic fungal pathogen which also causes disease on apples, pears, citrus fruits, strawberries, tomatoes, tobacco, blueberry and several other plants [14,15]. Leaf spot disease caused by *Alternaria alternata* is becoming a limiting factor and posing a major problem in chilli production [16]. Alternaria leaf spot is a widespread and highly destructive disease that infects chilli plants, and yield loss caused by these diseases has been recorded up to 100% under congenial environment conditions [17]. Yadav et al. (2015) reported the leaf spot disease in chilli caused by *Alternaria alternata* to be a highly destructive and major cause of loss in its yield [18].

Studying the effects of environmental factors on the biological activities of extracts and their bio formulations will help to improve their storage condition and maintenance of their efficacy in the presence of influencing physical factors for a prolonged period. The stability of plant-based extracts and their bio-formulations with respect to various temperature and pH treatments has been studied by Bonjar, and Farrokhi [19]. Rangnathan and Balajee, (2000) investigated the effects of different physical factors on the biological activities of ethanolic extract of *Ocimum sanctum* and *Cassia alata* [20]. The extract of black mulberry fruits *Morus nigra* possesses a high amount of antioxidant activity. Issa et al. (2013) investigated the effect of heat, pH and storage time on the stability of fruit extract of *Morus nigra* and its antioxidant activity [21]. Hedayat et al.*,* (2010) reported that temperature treatments at 40 °C, 30 °C, 60 °C and 100 °C did not affect the antimicrobial activity of the chloroform extract of *Polygonum avivculare* whereas higher activity was reported at acidic pH 2–6 [22]. The authors also reported a reduction in antimicrobial activity at alkaline pH. Baitha et al. (2020) reported the stability of bioactive components present in the ethanolic extract of *Syzygium aromaticum* over a wide range of pH and temperature values [23].

Cows in India are extremely similar to how cow excreta (dung and urine) have a holistic view in Hindu mythology and have demonstrated antibacterial efficacy from ancient times. Rajeswari et al. discovered the potent antifungal effects of cow dung extract on *Klebsiella pneumonia* and *Escherichia coli* [24]. The substantial action of cow dung and urine against the phytopathogenic fungus *Colletrotrichum falcatum* was also demonstrated by Krunal et al. (2017) [25].

To investigate the phytotoxic effects of *Lawsonia* leaf extracts on chilli, we conducted a pro-glass experiment as well as an in vitro seed germination and seedling growth bioassay. Our ultimate objective is to create appropriate ways for establishing *Lawsonia inermis* extracts as a safe and cost-effective biocontrol alternative to conventional fungicides.

Determination of the stability of antifungal potential of these extracts and their bio formulations is important for the establishment of their commercial utility and viability in real agricultural field conditions. Hence in the present study, the antifungal activity of 100% alcoholic crude extract, partially purified acetone extract and four different bio formulations has been evaluated under exposure to different physical factors viz. sunlight, temperature, pH and storage period.

## 2 Materials and methods

In the present study effect of various physical factors on the antifungal activity of screened bio-formulations has been determined as well as checked for phytotoxicity against germination and seedling growth of host (Chilli). The selected four bio-formulations were treated with varying ranges of sunlight, temperature, pH and storage period. The effect of physical factors was studied as a function of change in the antifungal activity of biomaterials.

### 2.1 Preparation of crude extract

The crude extract was made using Shadomy and Ingraff's modified cold extraction technique in 1989. Water, 50% hydro alcohol, and 100% alcohol were used to make the cold extract. For 48 h, 20 g of dried and powdered plant material was immersed in 100 ml of solvent (alcohol, water, and 50% hydro alcohol). The decoction was filtered using Whatman filter paper no. 1 and then vacuum dried using a rotary vacuum evaporator. The solvent was recycled and the dried residue was utilized as a crude extract.

### 2.2 Preparation of partially purified extract

The extract was partially purified using the hot extraction process, which comprises several solvent extractions to separate distinct phytochemical ingredients from plant segments [26]. The solvent series used for successive separation was nonpolar to polar *i.e.* Petroleum ether → Benzene → Chloroform → Acetone → Alcohol →Methanol →and Water. Continuous extraction of powdered dried plant material in a soxhlet device with the aforesaid sequence of organic solvents is used in this procedure. After each extraction with the next solvent, the plant material was dried in an oven at temperatures below 50 °C. Due to its inhibitory efficacy against the test pathogen, only the acetone fraction is employed in this study for phytotoxicity testing. 40 g of dry plant powder were placed in a soxhlet extraction device and extracted with 280 ml of acetone until all benzene soluble fractions were removed. The extract was vacuum dried in the vacuum evaporator. Before the phytotoxicity assessment, crude and partly purified extracts were tested for solubility in DMSO, DMF, Acetone, Methanol, Alcohol, and water. We employed a solvent with a higher solubilizing ability for maximal extracts in our further investigation. After the crude and partially purified extracts have dissolved in their respective solvents, residue remains. The extract that was more soluble in the solvent tended to leave less residue. The determination of residual % was done by the following formula

% Residue (A) = (a)/ (b) × 100

Where, (a) = weight of residue of crude extract, (b) = weight of crude extract.

### 2.3 Preparation of bio-formulation

In vitro antifungal activity against *Alternaria alternata* was tested using bio-formulations made by mixing active fractions of plant extracts, elicitors, and binders. The most active plant extracts, such as 100% alcohol crude extract, partly purified acetone extract, and the best active elicitor and binder, neem oil cake and cow dung were blended in various combinations to create a total of 30 bio-formulations.

Plant extracts and bio-formulations were tested for efficacy over a period of time using a variety of physical conditions including heat, temperature, pH, and sunlight. The minimum inhibitory concentration (MIC) of partly purified extract, the efficacy of crude extract, and the efficacy of several bio-formulations against the test organism were all measured. In each series of trials against *Alternaria alternata*, a tube containing the minimal inhibitory concentration of extract, bio-formulation, and without extract/bio-formulation was kept as a control. The experiments in this investigation included a % alcohol crude extract and a partly purified alcohol extract of *Lawsonia inermis* leaves, as well as four of the most potent bio-formulations. Components of bio-formulation were used in the following ratios:

F1 Bio-formulation: (100% alcohol crude extract (6 ml): 100% Neem oil cake (2 ml): 100% cow dung (2 ml).

F2 Bio-formulation: (100% alcohol crude extract (3 ml): 100% Neem oil cake (4 ml): 100% cow dung (3 ml)).

F3 Bio-formulation: (Partially purified acetone extracts (3 ml): 100% Neem oil cake (3 ml): 100% cow dung (4 ml).

F4 Bio-formulation: (Partially purified acetone extracts (4 ml): 100% Neem oil cake (3 ml): 100% cow dung (3 ml).

### 2.4 Phytotoxicity assay

The conventional blotter method was used to assess the phytotoxicity of crude and partly purified extracts on seed germination and seedling growth in chilli [27]. Pre-sterilized chilli seeds were soaked in 5 mg/ml, 10 mg/ml, 15 mg/ml, 20 mg/ml and 25 mg/ml extract concentration respectively for 30 min and kept on moistened Whatman no.1 filter paper in sterilizes Petri plates. at 27 °C. Seeds were placed on a labelled Petri plate. Sterile distilled water was used at the place of extract for control. Several germinated seeds were recorded for different time intervals and radical length was measured every 24 h up to 9 days. The germination percentage of seeds was calculated by the following formula:

Germination % = Total germinating seeds / Total treated seeds x100

### 2.5 Effect of physical factors on the antifungal activity of bio formulations

#### 2.5.1 2.5.1Effect of sunlight

The effect of sunlight on the viability of extracts and bio-formulation was studied according to the method suggested by Wang & Ke-Qiang, (2001) [28]. Pre-sterilized chilli seeds were soaked for 30 min in extract concentrations of 5 mg/ml, 10 mg/ml, 15 mg/ml, 20 mg/ml, and 25 mg/ml and maintained on moistened Whatman no.1 filter paper on sterilized Petri plates. The effect of the extract and bio-formulation on effectiveness was then tested using the tube dilution method and the poison food methodology. The poison food procedure involved pouring 18 ml of molten PDA medium into test tubes, which were then autoclaved. Following the solidification of the molten sterilized medium, a 6 mm inoculum disc of a 7-day-old fungal culture was aseptically injected upside down in the centre of the Petri plate and incubated at 25 ± 2 °C.

On the seventh day of incubation, the average diameter of the fungal colonies was determined, and per cent mycelia growth inhibition was calculated using the formula below.

$$\text{Mycelial}\;\text{growth}\;\text{inhibition}=\frac{gc–gt}{gc}\times 100$$

Where, gc = growth of control, gt = growth of in treatment.

#### 2.5.2 Effect of heat

The efficacy of the extract and bio-formulation was determined using the approach proposed by Rath et al. (2001) [29]. The effects of dry heat were investigated by placing sterile glass vials containing 100% alcoholic crude extract, partly purified acetone extract, and four distinct bio formulations (F1, F2, F3, F4) in a hot air oven for 4 h at two different temperatures: 40 °C and 90 °C. Similarly, the effect of wet heat was evaluated by placing the same set of vials in a water bath for 4 h at 50 °C and 100 °C temperatures, respectively. The set of vials placed at room temperature (untreated) served as control. Afterwards using the poison food technique, the percent mycelial inhibition was measured.

#### 2.5.3 Effect of pH

The effect of pH on the antifungal efficacy of extracts and bio formulations was studied after the method of Dixit et al. (1981) [30]. The original pH of the leaf extract and bio formulations was 7. 0 which was altered to pH 4 and pH 9 using the 0.1 N HCl and 0.1 NaOH, respectively. Sterile PDA media was added into the tubes containing extract and bio formulations and further inoculated with *Alternaria alternata*. After inoculation, the tubes were incubated for 72 h at 28 ± 1 °C temperature. After completion of incubation, the tubes were observed for change in MIC (Minimum Inhibitory Concentration) of bio formulation and extracts.

#### 2.5.4 Effect of the storage period

To evaluate the effect of storage time on the bio-control efficacy of extract and bio formulation a recommended method by Rath et al. (2001) [29]. was used. Extract and bio formulations were stored at room temperature maximum for 12 months. Using the two-fold serial dilution approach and the poison food technique, the change in their antifungal activity was assessed at regular intervals of 6 months and a maximum up to 12 months.

### 2.6 Statistical analysis

All the experiments were performed in triplicates, repeated thrice via a randomized design. The obtained experimental data were statistically analyzed with IBM SPSS Statistics Ver. 20 software. The statistical data were expressed as the average of three independent replications ± standard error (SE).

## 3 Results and observations

Table 1, Table 2, Table 3, Table 4, Table 5, Table 6, Table 7, Table 8 show the effects of various physical conditions such as sunlight, heat, pH, and long-term storage on the extract and bio-formulation of *Lowsonia inermis* leaf. Direct sunlight exposure for 15 and 30 h had no influence on the effectiveness of acetone extract and bio-formulations, as shown in Table 1, Table 2 In the instance of a 100% alcohol crude extract, 15 h of exposure showed no impact, while 30 h of exposure resulted in a modest drop-in activity. The effect of wet and dry heat on extract and bio-formulation effectiveness is shown in Table 3, Table 4 Up to 50 °C of wet heat and 40 °C of dry heat, there was no effect on the activity of 100% alcoholic crude extract and bio-formulation ratio F1, F2, F3, F4. However, heating at 100 °C of wet heat and 90 °C of dry heat for 4 h resulted in a slight decrease in extract and bio-formulation efficacy as a slight growth of test fungus was observed.

**Table 1:** Effect of Sunlight Exposure on Crude and partially Purified Extract of *Lawsonia inermis* Leaf Extract against *Alternaria alternata* (Growth Diameter mm).

S. No.	Extracts	Unexposed condition	15 h	30 h
1.	100% Alcoholic Crude extract	33.66 ± 0.01 (mm)	33.66 ± 0.01 (mm)	33 ± 0.01 (mm)
2.	Partially purified extract (Acetone)	1.25 mg/ml	1.25 mg/ml	1.25 mg/ml
3.	Control (Without Extract)	82.33 ± 0.52 mm

**Table 2:** Effect of Sunlight Exposure on Bio-formulation against *Alternaria alternata* (Growth Diameter mm).

	Bioformulation	Unexposed Condition	15 h	30 h
1.	F1	16.6 ± 0.52	16.6 ± 0.52	16.6 ± 0.52
2.	F2	16.66 ± 0.52	16.66 ± 0.52	16.66 ± 0.52
3.	F3	15.33 ± 0.15	15.33 ± 0.15	15.33 ± 0.15
4.	F4	16 ± 0.23	16 ± 0.23	16 ± 0.23
5.	Control (Without Extract)	82.33 ± 0.52 mm

**Table 3:** Effect of Heat on Crude and Partially Purified Extract of *Lawsonia inermis* Leaf against *Alternaria alternata* (Growth Diameter mm).

S. No.	Extract	Wet Heat		Dry Heat	
		50 °C	100 °C	40 °C	90 °C
1.	100% Alcoholic	29.66 ± 0.01 (mm)	30 ± 0.01 (mm)	29.66 ± 0.01 (mm)	25.66 ± 0.01 (mm)
2.	Partially Purified Acetone	1.25 mg/ml	Slight growth	1.25 mg/ml	Slight growth
3.	Control (Without Extract)	82.33 ± 0.52

**Table 4:** Effect of Heat on Bio-formulation against *Alternaria alternata* (Growth Diameter mm).

S. No.	Bio-formulation	Wet Heat		Dry Heat	
		50 °C	100 °C	40 °C	90 °C
1.	F1	16.6 ± 0.52	20.6 ± 0.52	16.6 ± 0.52	22.6 ± 0.52
2.	F2	16.66 ± 0.52	20.66 ± 0.52	16.66 ± 0.52	20.66 ± 0.52
3.	F3	15.33 ± 0.15	23.33 ± 0.15	15.33 ± 0.15	20.33 ± 0.15
4.	F4	16 ± 0.23	21 ± 0.23	16 ± 0.23	21 ± 0.23
5.	Control (Without Extract)	82.33 ± 0.52

**Table 5:** Effect of pH on Crude and Partially Purified Extract of *Lawsonia inermis* leaf against *Alternaria alternata* (Growth Diameter mm).

S. No.	Extract	Control (pH 7)	pH 4	pH 9
1.	100% Alcoholic crude	29.66 ± 0.01 (mm)	30 ± 0.01 (mm)	30 ± 0.01 (mm)
2.	Partially Purified Acetone fraction	1.25 mg/ml	Slight Growth	Slight Growth
3.	Control (Without Extract)	82.33 ± 0.52

**Table 6:** Effect of pH on Bio-formulation against *Alternaria alternata* (Growth Diameter mm).

S. No.	Bio-formulation	Control (pH 7)	pH 4	pH9
1.	F1	16.6 ± 0.52	22.6 ± 0.52	22.6 ± 0.52
2.	F2	16.66 ± 0.52	20.66 ± 0.52	20.66 ± 0.52
3.	F3	15.33 ± 0.15	20.33 ± 0.15	20.33 ± 0.15
4.	F4	16 ± 0.23	21 ± 0.23	21 ± 0.23
5.	Control (Without Extract)	82.33 ± 0.52

**Table 7:** Effect of Storage period on Crude and Acetone Extract of Leaf against *Alternaria alternate* (Growth Diameter mm).

S. No.	Extract	Fresh Extract	6 Months	12 Months
1.	100% Alcoholic crude	29.66 ± 0.01 (mm)	29.66 ± 0.01 (mm)	29 ± 0.01 (mm)
2.	Partially Purified Acetone	1.25 mg/ml	1.25 mg/ml	1.25 mg/ml
3.	Control (Without Extract)	82.33 ± 0.01 (mm)

**Table 8:** Effect of Storage period on Bio-formulation against *Alternaria alternata* (Growth Diameter mm).

S. No.	Bio-formulation	Fresh Extract	6 Months	12 Months
1.	F1	16.6 ± 0.52	16.6 ± 0.52	16.6 ± 0.52
2.	F2	16.66 ± 0.52	16.66 ± 0.52	16.66 ± 0.52
3.	F3	15.33 ± 0.15	15.33 ± 0.15	15.33 ± 0.15
4.	F4	16 ± 0.23	16 ± 0.23	16 ± 0.23
5.	Control (Without Extract)	82.22 ± 0.01 (mm)		

The effect of altering pH on the efficiency of plant extract and bio-formulation is shown in Table 5, Table 6 The inhibitory impact on extract and bio-formulation efficacy was at neutral and alkaline pH up to 9, although there was a reduction in antifungal activity against *Alternaria alternata* at acidic pH (pH 4).

The effect of long-term storage of extract and bio-formulation at room temperature is shown in Table 7, Table 8 The effectiveness of the extract and bio-formulation was unaffected by storage for 6 and 12 months, and the antifungal activity was comparable to that of fresh extract.

## 4 Discussion

In comparison to conventional pesticides, bio-formulation utilizing plant extracts is effective in the control of numerous illnesses since it has no negative effects. Only when bio-formulations can retain stability and when physical conditions have no effect on their activity can they be considered viable. Physical variables such as pH, temperature, and sunlight exposure can impact the antimicrobial properties of extracts and bio-formulations. Because all of these variables are responsible for a change in the chemical composition of molecules that have antimicrobial activity.

The antifungal activity of acetone extract and bio-formulation did not change after being exposed to direct sunlight, indicating that the active components in acetone extract and bio-formulation are light stable and do not suffer photo-oxidation. The antifungal activity of a 100% alcoholic crude extract was also maintained after 15 h of exposure to sunlight. Similar findings were reported by Wang & Ke-Qiang (2001) [28]. The active compounds in the acetone extract of *Lawsonia inermis* that have antifungal potential are unlikely to be destroyed by sunlight.

The effect of heat on a 100% alcohol crude extract and bio-formulations F1 and F2 revealed that the active principles may tolerate wet and dry heat up to 50 and 40 °C, respectively. While lengthened exposure to 100 °C wet heat and 90 °C dry heat reduced the extract's antifungal potential, acetone extract and bio-formulation F3 and F4 were unaffected. Singh et al. (2006) came to the same conclusion on *Foeniculum vulgare* volatile oil and its acetone extract's antifungal and antioxidative properties. Magdy et al. (2014) also found that temperatures of 4 °C, 30 °C, 60 °C, and 90 °C had no effect on the activity of plant extracts from *Cinnamomum cassia, Alium sativum, Syzygium aromaticum, Punica granatum*, *Citrus lemoniumn*, and *Hibiscus sabdariffa* [31]. The temperature resistance assays reveal that the phytoconstituents are thermostable, but heating them to 120 °C or above reduces or eliminates their antibacterial action. The temperature resistance assays reveal that the phytoconstituents are thermostable, but heating them to 120 °C or above reduces or eliminates their antibacterial action.

The antifungal activity of *Lawsonia inermis* leaf extract and bio-formulation was found to be stable at pH 7 and 9. At pH 4, the activity of the same was found to be reduced. These findings show that the extract's active principles are more active at neutral pH. Yen and Duh (1993) reported that a methanol extract from peanut hulls had a higher antioxidant activity at neutral and acidic pH [32]. Doughari, (2006) found an increase in phytoconstituent activity in the presence of an acidic medium [26].

Jeffery (2006) also explores the influence of other physical parameters on the antibacterial activity of pepper leaf extracts, such as heat and temperature [33]. According to Arabshahi et al. (2007), the antioxidant activity of extracts of mint leaves, carrots, and drumsticks changes with pH [34]. Srinivasan et al. (2009) found that when the pH value increased, the antibacterial activity of *Allium sativum* extract decreased, peaking at pH 9 [35]. According to Bayliak et al. (2016), antioxidant activity of aqueous extracts of *Rosa canina, Rhodiola rosea, Hypericum perforatum,* and *Gentiana lutea* decreases with storage [36]. However, the results of the studies suggest that long-term storage has no effect on the efficacy of the extract and bio-formulation.

During storage, many physical factors increase the ageing of plant extracts and the chemical degradation of active components, resulting in a reduction in antifungal activity. Because the degree of changes in biological activity and chemistry of secondary metabolites after storage varies by species, these effects differ [37].

Similar findings about various physical factors and their effect on antifungal activity of extract have also been reported by several workers [13,20,31,34,[38], [39], [40], [41], [42]]. They also hypothesized that changes in chemical composition might be caused by environmental factors such as temperature, sunlight, heat, and long-term storage. The amount of active ingredients in extracts may decrease as a result of these changes, and the magnitude of change in phytochemical contents of extracts differs between species.

## 5 Conclusion

Recent research found that plant-based fungicides are suitable alternatives to chemical fungicides. Various physical factors can affect the antifungal activity of plant extract in actual agricultural field conditions. Thus, detailed investigations are required on the effect of various physical factors on the antifungal efficiency of plant extracts before their application in the agriculture field. The present study investigated the effect of sunlight exposure, dry and wet heat, pH and storage time on the antifungal activity of *Lawsonia inermis* leaf extracts and bio-formulation. Maximum stability in antifungal activity was found with the partially purified acetone extract. However, the crude and alcohol extract and bio-formulation showed a mild reduction in activity at higher temperature ranges viz. 90 °C and 100 °C. Similarly, a shift in pH at 4 and 9 caused a slight reduction in the activity of crude alcohol extract and bioformulation. The antifungal activity of all the studied materials remains unaltered for up to 12 months of storage. Constant antifungal activity of acetone extract under varying physical factors proved its importance in field conditions hence it can be further used as a suitable alternative to chemical fungicides without posing any serious hazards to the environment and human health.

## Declaration of competing interest

The authors declare no interest of conflict.

## Data Availability

Data will be made available on request.
